# Supplementary material for: Comparative multiomics analysis of cell physiological state after culture in a basket bioreactor
Source: Sci Rep. 2022 Nov 23;12:20161. doi: 10.1038/s41598-022-24687-4 (PMC9686226; doi:10.1038/s41598-022-24687-4)
Supplement: Supplementary file 1 — Supplementary Information 1. [file 41598_2022_24687_MOESM1_ESM.zip › raw data/Transcriptomic raw data/6.Enrichment/1.GO/TE1vsTC1/DOWN/TE1vsTC1.down.GOmf_DAG.pdf]

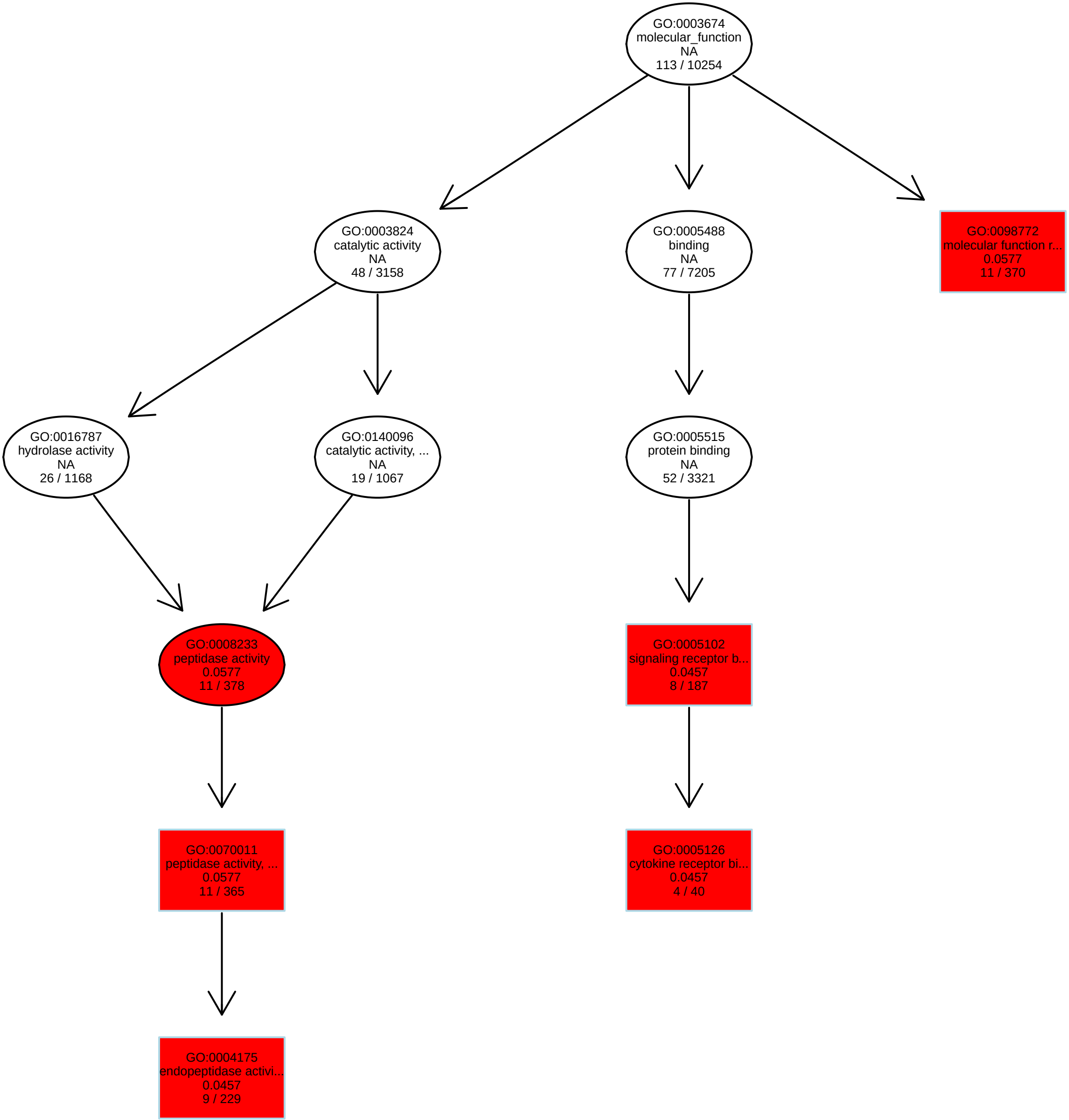
GO:0003674
molecular_function
NA
113 / 10254
GO:0003824
catalytic activity
NA
48 / 3158
GO:0005488
binding
NA
77 / 7205
GO:0098772
molecular function r...
0.0577
11 / 370
GO:0016787
hydrolase activity
NA
26 / 1168
GO:0140096
catalytic activity, ...
NA
19 / 1067
GO:0005515
protein binding
NA
52 / 3321
GO:0008233
peptidase activity
0.0577
11 / 378
GO:0005102
signaling receptor b...
0.0457
8 / 187
GO:0070011
peptidase activity, ...
0.0577
11 / 365
GO:0005126
cytokine receptor bi...
0.0457
4 / 40
GO:0004175
endopeptidase activi...
0.0457
9 / 229
